# Lobular Breast Cancer: Histomorphology and Different Concepts of a Special Spectrum of Tumors

**DOI:** 10.3390/cancers13153695

**Published:** 2021-07-22

**Authors:** Matthias Christgen, Gábor Cserni, Giuseppe Floris, Caterina Marchio, Lounes Djerroudi, Hans Kreipe, Patrick W. B. Derksen, Anne Vincent-Salomon

**Affiliations:** 1 Institute of Pathology, Hannover Medical School, Hannover, Carl-Neuberg-Str. 1, 30625 Hannover, Germany; Kreipe.Hans@MH-Hannover.de; 2 Department of Pathology, University of Szeged, 6725 Szeged, Hungary; cserni@freemail.hu; 3 Department of Pathology, Bács-Kiskun County Teaching Hospital, 6000 Kecskemét, Hungary; 4 Department of Pathology, UZ Leuven Gasthuisberg Campus, Universitair Ziekenhuis Leuven, Pathology, Herestraat 49, 3000 Leuven, Belgium; giuseppe.floris@uzleuven.be; 5 Department of Medical Sciences, University of Turin, 10124 Turin, Italy; caterina.marchio@unito.it; 6 Unit of Pathology, Candiolo Cancer Institute, FPO IRCCS, 10060 Candiolo, Italy; 7 Department of Diagnostic and Theranostic Medicine, Department of Pathology, Institute Curie, PSL-Research University, 26 rue d’Ulm, 75005 Paris, France; lounes.djerroudi@curie.fr; 8 Department of Pathology, University Medical Center Utrecht, Heidelberglaan 100, 3584 CX Utrecht, The Netherlands; P.W.B.Derksen@umcutrecht.nl

**Keywords:** LCIS, LIN, pleomorphic, solid, tubulolobular, HER2, beta-catenin, p120-catenin

## Abstract

**Simple Summary:**

Invasive lobular breast cancer (ILC) is a special type of breast cancer (BC) that was first described in 1941. The diagnosis of ILC is made by microscopy of tumor specimens, which reveals a distinct morphology. This review recapitulates the developments in the microscopic assessment of ILC from 1941 until today. We discuss different concepts of ILC, provide an overview on ILC variants, and highlight advances which have contributed to a better understanding of ILC as a special histologic spectrum of tumors.

**Abstract:**

Invasive lobular breast cancer (ILC) is the most common special histological type of breast cancer (BC). This review recapitulates developments in the histomorphologic assessment of ILC from its beginnings with the seminal work of Foote and Stewart, which was published in 1941, until today. We discuss different concepts of ILC and their implications. These concepts include (i) BC arising from mammary lobules, (ii) BC growing in dissociated cells and single files, and (iii) BC defined as a morpho-molecular spectrum of tumors with distinct histological and molecular characteristics related to impaired cell adhesion. This review also provides a comprehensive overview of ILC variants, their histomorphology, and differential diagnosis. Furthermore, this review highlights recent advances which have contributed to a better understanding of the histomorphology of ILC, such as the role of the basal lamina component laminin, the molecular specificities of triple-negative ILC, and E-cadherin to P-cadherin expression switching as the molecular determinant of tubular elements in *CDH1*-deficient ILC. Last but not least, we provide a detailed account of the tumor microenvironment in ILC, including tumor infiltrating lymphocyte (TIL) levels, which are comparatively low in ILC compared to other BCs, but correlate with clinical outcome. The distinct histomorphology of ILC clearly reflects a special tumor biology. In the clinic, special treatment strategies have been established for triple-negative, HER2-positive, and ER-positive BC. Treatment specialization for patients diagnosed with ILC is just in its beginnings. Accordingly, ILC deserves greater attention as a special tumor entity in BC diagnostics, patient care, and cancer research.

## 1. Introduction

Modern tumor classifications are based on histomorphology enriched with molecular analyses that confirmed the taxonomic network of tumor entities established by histomorphology [1,2]. Clinical “basket trials” with multiple cancer types have shown that histological entities determine the clinical behavior of tumors to a degree not predicted by preclinical models [3].

Histomorphologic assessment of surgical tumor specimens developed in the 19th century. A first classification of breast tumors was proposed by Cheatle and Cutler in 1930 [4]. This classification described breast cancers (BCs) arising in different anatomic compartments, such as main ducts and lobules [4]. Histopathologists had noted a distinct kind of BC arising from a “pre-cancerous” epithelial proliferation confined to mammary lobules. Early microscopic illustrations were provided by Cornil in 1908 (reprinted in Rosen et al., 1978) [5,6]. The term “lobular carcinoma” was eventually coined by Stewart and Foote in 1941 [7]. They were the first to emphasize the loss of cell cohesion as the key histological feature of lobular carcinoma in situ (LCIS) and invasive lobular breast cancer (ILC) [7]. In 1975, Rosen et al. reported that ILC is estrogen receptor (ER)-positive [8]. Loss of the cell adhesion molecule E-cadherin was first described by Moll et al. and Gamallo et al. in 1993 [9,10,11,12]. Subsequent genetic studies in mice showed that E-cadherin loss causes ILC development and progression [13]. It is now well established that mutational inactivation of E-cadherin is an oncogenic driver in ILC [14,15,16,17,18].

The world health organization (WHO) classification of tumors of the breast (5th edition from 2019) recognizes ILC as the most common special type of BC [19]. ILC accounts for 10–15% of all BC cases [20]. ILC is less common in Asian populations (2–6%) [21,22,23]. ILC is associated with higher patient age, higher pT stage, higher nodal stage, lower histological grade, and is over-represented in bilateral and primary metastatic BC [20,24,25]. ILC is associated with a distinct pattern of metastatic dissemination. Metastasis to the digestive tract, ovaries, bones, leptomeninx, orbital soft tissue, and skin occurs more often in ILC [24,26,27,28,29,30,31,32,33,34]. The rate of multiple metastases is higher in ILC compared to other BCs [35]. Pathological complete response to neoadjuvant chemotherapy is rare and resection margins are more often positive [20,36,37,38]. ILC differs from BC of no special type (NST, formerly known as ductal BC) with respect to DNA copy number (CN) alterations, gene expression profiles, mutational characteristics, and tumor microenvironment (immune cells and cancer-associated fibroblasts) [39,40,41,42,43,44,45,46].

This review focuses on the histomorphology of ILC. We recapitulate developments in the histomorphologic assessment of ILC from its beginnings until today. We discuss different concepts of ILC and their implications and provide an overview of published histologic ILC variants. Last but not least, we highlight recent advances, which have contributed to a better understanding of this special tumor entity.

## 2. Concepts and Perceptions of ILC and Their Influence on BC Diagnosis

### 2.1. ILC as Carcinoma Arising in Lobules

**Figure 1:**
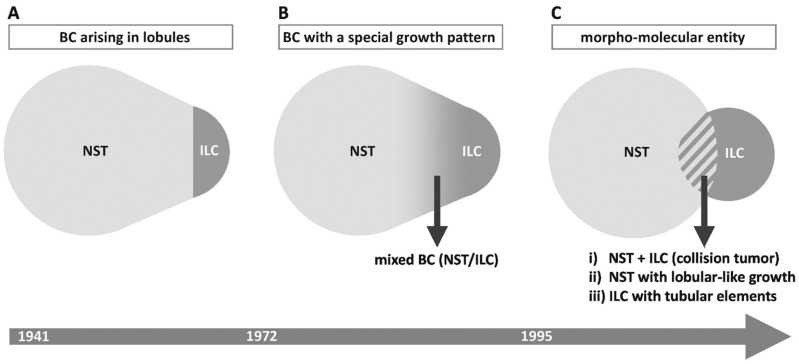
Different concepts of ILC as a special tumor entity and their implications. This is a Venn diagram-like schematic presentation of the relation between BC of NST and ILC, according to different concepts. (**A**) ILC as BC arising in lobules and terminal ducts (Foote and Stewart in 1941) [7]. BC of NST and ILC are clearly separated (based on the presence or absence of LCIS). For details, see text Section 2.1. (**B**) ILC as BC with a special growth pattern (1972 until today, consistent with the 5th edition of the WHO classification of tumors) [19,56]. The boundary between BC of NST and ILC is less clearly defined compared to concept “A”. BCs with indefinite histomorphology may be classified as mixed BC NST/ILC. For further details, please see text Section 2.2 and Appendix A Table A1. (**C**) ILC as a morpho-molecular entity (a concept unofficially prevalent in clinical research since the discovery of *CDH1* mutations in 1995) [11]. BC of NST and ILC share a morphologic overlap. Cases in the morphologic overlap may be interpreted as (i) collision tumors, (ii) NST with lobular-like growth pattern but no loss of cell adhesion, and (iii) ILC with tubular elements [58,59]. For details, see text Section 2.3. An approximate time scale showing the origin of these concepts is given at the bottom.

The concept of ILC has been changing over time (Figure 1A). In 1941, Foote and Stewart defined ILC as BC arising in lobules and terminal ducts, characterized by loss of cell cohesion in the in situ tumor component, and characterized by loose, isolated cells in the invasive tumor component [7,47]. LCIS was a prerequisite for the diagnosis ILC. In the 1960s, ILC accounted for 5% of BCs, and all cases were associated with LCIS [48]. Today, ILC accounts for 10–15% of BCs, and ILC is associated with LCIS in approximately 50% of cases [49,50,51]. The original definition of ILC provided a straightforward distinction between lobular and non-lobular BC based on the presence or absence of LCIS. However, the frequency of ILC was underestimated, because ILC without LCIS was not classified as such. Foote and Stewart then asserted a “high probability” of a lobular origin for BCs growing in loose, isolated cells, even if LCIS could not be proven by microscopy [7]. This seemed particularly relevant for ILC in atrophic breast tissue with few lobules in elderly patients. Single pagetoid tumor cells in terminal ducts were proposed as the earliest form of LCIS and as a possible origin of ILC in atrophic breast tissue [7]. Of note, ILC can even arise in the human male breast and in mouse mammary glands, which are generally lacking lobules [52,53,54,55]. However, LCIS remained a prerequisite for the diagnosis of ILC until the 1970s [56]. Foote and Stewart were skeptical whether or not ILC is a separate tumor entity, beyond its anatomic relation to mammary lobules. Foote and Stewart passed away in 1989 and 1991, respectively [57]. They did not have the chance to witness the discovery of *CDH1/*E-cadherin mutations in ILC. It remains unknown how they would have re-considered ILC in the light of this finding.

### 2.2. ILC as Carcinoma Growing in Single Files

The perception of ILC changed in the 1970s (Figure 1B). In 1972, Fechner reported a series of BCs, which appeared to be invasive lobular BCs but lacked LCIS. However, LCIS was identified in the contralateral mastectomy specimens. Fechner concluded that LCIS had probably been present in both breasts, but had been overgrown by invasive tumor cells in the ipsilateral mammary glands [56]. Accordingly, he classified these cases as ILC without LCIS [56]. This work rendered the diagnosis of ILC independent from LCIS. In his book “Problems in breast pathology”, Azzopardi likewise stressed the fact that the overarching pathological distinction between lobular BC and ductal BC depends on cyto-architectural features rather than on the precise site of origin [60]. LCIS is an optional feature since that time. Remaining criteria for the diagnosis of ILC included small, loose tumor cells and growth in single files, features that are still valid today [19,56]. The current WHO classification defines ILC as BC composed of dyscohesive cells that are mostly individually dispersed or arranged in single files [19]. However, a large proportion of ILCs does not show this classic growth pattern [43,46,61,62,63,64,65,66]. In 1975, Fechner reported an ILC variant with solid growth and description of further variants followed [66]. Furthermore, ILC and BCs of NST constitute a morphological continuum with a proportion of indefinite cases termed mixed BC (NST/ILC) [67]. Mixed BC (NST/ILC) accounts for up to 18% of BC cases (Appendix A, Table A1) [42,61,67,68,69,70,71]. According to the traditional WHO definition, this term was applicable for BCs showing a mixture of 11–50% ductal and 50–89% lobular growth pattern [72]. According to the revised WHO definition, this term is applicable for BCs showing a mixture of 10–90% BC of NST and 10–90% lobular subtype [19]. Loss of E-cadherin in the lobular tumor component is not a formal requirement. In research studies, most investigators exclude mixed BC (NST/ILC) from further analyses [68,69,70,71,73]. The WHO classification provides clear guidelines for the classification of BC as NST or ILC. However, the assignment of a growth pattern to either ductal or lobular or mixed type (NST/ILC) remains to some extent subjective. For instance, BC of NST can grow in slender trabeculae, which mimic single files, but tumor cells have not lost cohesion [74]. Interobserver agreement is variable in this constellation [75,76,77]. Two large clinical trials (MINDACT and WSG PlanB trials) have recently reported the results of central pathology review of thousands of BC specimens, which confirmed ILC in 60% (395/654) and in 66% (253/385) of cases classified as ILC by local assessment. Conversely, central review changed the diagnosis of NST to ILC in only 2% (37/1763) of cases [61,78]. Hence, ILC might be slightly over-diagnosed in current practice and according to the concept of a special growth pattern.

### 2.3. ILC as a Morpho-Molecular Entity

Alternatively, ILC can be considered as a morpho-molecular BC entity (Figure 1C). In 1995, somatic mutational inactivation of *CDH1/*E-cadherin was discovered in ILC [11,12]. Subsequently, LCIS and adjacent ILC were shown to harbor identical *CDH1* mutations [79,80,81,82]. This and other molecular genetic analyses verified the clonal relatedness of LCIS and ILC [79,82,83,84,85]. Inactivation of *CDH1*/E-cadherin provided a molecular explanation for the key histological feature of ILC, namely, the loss of cell cohesion. Most *CDH1* mutations are somatic frameshift or nonsense mutations resulting in truncated, non-functional E-cadherin proteins [43]. These mutations are typically accompanied by loss of the remaining wild-type *CDH1* allele on chromosome 16q22.1 (detectable as loss of heterozygosity, LOH). Mutant *CDH1* transcripts are also downregulated by nonsense-mediated mRNA surveillance mechanisms [86]. In a subset of ILC cases, loss of E-cadherin is not associated with a detectable *CDH1* mutation. This has been explained by epigenetic silencing and/or transcriptional repression, but the relevance of these proposed alternative mechanisms has remained controversial [42,87,88]. Lack of a detectable *CDH1* mutation may also be related to insufficient sequencing depth or low tumor cellularity [40,89]. In some instances, *CDH1* missense mutations, in-frame deletions, and even truncating mutations can be associated with preserved E-cadherin expression [43,90]. The proportion of *CDH1*-mutant/E-cadherin-positive ILC varies in the literature, and the varying figures are possibly related to different anti-E-cadherin antibodies used for immunohistochemistry (IHC). In the series of Desmedt et al., immunoreactivity for the anti-E-cadherin antibody NCH-38 was retained in 8/156 (5%) ILCs harboring *CDH1* mutations [43]. In the series of Grabenstetter et al., immunoreactivity for the anti-E-cadherin antibody clone-36 was retained in 47/202 (23%) BCs harboring somatic *CDH1* mutations [91]. E-cadherin forms the core of the adherens junctions (AJs), structures that provide the cell–cell contacts in which cadherins bridge the neighboring plasma membranes via homophilic interactions and catenins bind to the actin cytoskeleton [92]. Rarely, AJ complex members other than E-cadherin, such as α-catenin/*CTNNA1,* are alternative mutation targets in ILC [93,94,95]. However, mutations in β-catenin/*CTNNB1*, which are not uncommon in various cancer entities and in some mesenchymal tumors [96,97,98], are consistently absent in ILC [43,99]. Whatever the mechanism, loss of E-cadherin is observed in 55–100% of cases (Appendix A, Table A2) [9,10,11,12,41,42,43,59,61,82,88,90,99,100,101,102,103,104,105,106,107,108,109,110,111,112,113,114,115,116,117,118,119]. *CDH1* mutation, as determined by DNA sequencing, is observed in 12–85% of cases (Appendix A, Table A2). The large range of reported *CDH1* mutation frequencies (12–85%) may have technical reasons, including different sequencing methods and different pre-processing (DNA sequencing with or without tumor microdissection). Moreover, slightly inconsistent histomorphologic subtyping may contribute to inconsistent *CDH1* mutations frequencies, at least to some extent. As mentioned above, >33% of BC cases initially diagnosed as ILC by local assessment were re-classified as non-lobular BC upon central re-review in two large clinical trials [61,78]. It may be relevant whether or not, or how often, E-cadherin IHC is utilized as an upfront ancillary method to support BC subtyping. It is likely that upfront E-cadherin IHC improves the accuracy with which *CDH1*-mutant BC is recognized and subsequently diagnosed as ILC. This may impact on *CDH1* mutation frequencies in ILC. Interestingly, the proportion of E-cadherin-negative and/or *CDH1*-mutant ILCs has been rising (Appendix A, Table A2). This may be due to improved sequencing techniques and/or may reflect an increased use of upfront E-cadherin IHC for initial BC subtyping. This appears to indicate that ILC is increasingly perceived as a morpho-molecular entity defined by combined morphological and molecular features related to impaired cell adhesion [74,120].

According to the experience of the authors of this review, it is not an uncommon opinion in the field of research that modern clinical trials would benefit from refined criteria for ILC, which are consistent with a morpho-molecular entity. However, this may be a point of disagreement among pathologists. Morphological features of ILC are well-established (see Section 3 and Section 4) and molecular features are well-described (see above and Section 3.3), but a precise, universally accepted definition of ILC based on a combination of both morphological and molecular items has remained a desideratum. According to the current WHO classification, loss of E-cadherin is a desirable but not a mandatory criterion [19]. As explained above, a small proportion of ILCs retain E-cadherin expression. However, these cases often harbor *CDH1* mutations that result in either truncation of the cytosolic tail of E-cadherin, or attenuation/inhibition of the homotypic in trans interaction of the extracellular E-cadherin domains [43,121]. In both instances, this results in a loss of cell adhesion or a dissociated growth pattern. Accordingly, a positive E-cadherin status does not exclude that a given BC is ILC. However, it should be noted that E-cadherin-positive ILCs often feature an aberrant, fragmented membranous, or diffuse perimembranous E-cadherin immunoreactivity (see Section 3.3) [74,109,122,123,124]. On the other hand, loss of E-cadherin can also occur in high grade basal-like (non-lobular) BCs, presumably during the later stages of tumor progression [125,126]. Hence, loss of E-cadherin per se is also not diagnostic for ILC. Recently described ILC classifier tools based either on artificial intelligence (AI)-based image analysis or on targeted DNA sequencing alone offer no practical solution [119,127]. In fact, they fail to bring together the morphological and molecular subdisciplines of BC diagnostics.

The authors of this review believe that a refined definition of ILC within a framework of a morpho-molecular entity could be achievable. One possible approach would require only two prerequisites: (i) assessment of the E-cadherin IHC status in all BCs considered as ILC based on histomorphology, and (ii) definition of key criteria that qualify a given BC as ILC, if E-cadherin expression is not entirely lost. Consequently, ILC might be defined as all BCs with a typical histomorphology (classic lobular growth pattern, variant lobular growth patterns) and an E-cadherin-negative status, plus all BCs with a typical histomorphology, an E-cadherin-positive status and one or more additional key criteria that overrule E-cadherin-positivity. Respective key criteria might be proposed and discussed among expert panels and may include (i) distinct phenotypic features (e.g., fragmented E-cadherin immunoreactivity, and/or aberrant expression of p120-catenin, or loss of β-catenin, which can stratify whether an observed E-cadherin expression represents a non-functional adherens junction, and can validate the ILC diagnosis [128]), (ii) distinct molecular features (e.g., mutation of *CDH1* or *CTNNA1* as determined by DNA sequencing, if available [95]), and (iii) distinct histological features (e.g., LCIS in multiple lobules within the tumor). However, such a refined algorithm for the diagnosis of ILC has not been formulated and is a desideratum, so far.

Irrespective of how to define ILC as a morpho-molecular entity in detail, it is interesting to note that this concept may influence the use of related diagnostic terms. By considering ILC as a distinctive morpho-molecular entity, which is a conceptual consideration, the term “mixed BC (NST/ILC)” may become problematic. This is because the traditional meaning of “mixed BC (NST/ILBC)” describes a growth pattern and is restricted to the morphological level. From the perspective of a morpho-molecular tumor entity, mixed-appearing BCs may rather be interpreted as an assortment of: (i) collision tumors or genetically divergent subclones (id est, NST plus ILC), (ii) BCs of NST with lobular-like growth pattern but no molecular alterations related to cell adhesion, and (iii) ILCs with tubular elements (Figure 1C) [58,59]. However, there is also evidence that BCs with mixed-appearing histomorphology are not always collision tumors, and this exemplifies that every concept has certain limitations [58,129].

In summary, current ILC definitions are often primarily restricted to morphological features rather than defining the entity within a morpho-molecular context.

## 3. Histomorphology

### 3.1. Classic ILC

**Figure 2:**
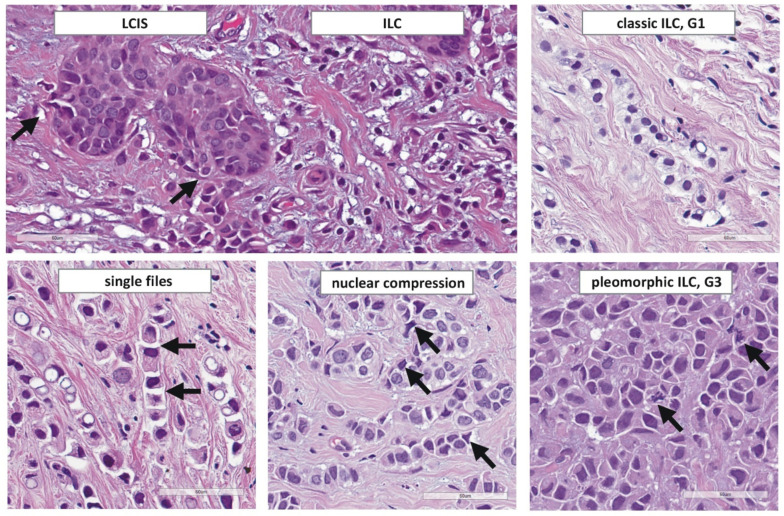
Histomorphology of ILC. From top left to lower right, photomicrographs (×400 magnification) illustrate: transition from LCIS to ILC (arrows indicate LCIS cells that breach through the basal lamina), classical ILC (G1), ILC arranged in single files with gaps between individual cells (arrows), ILC with prominent nuclear compression (arrows), pleomorphic ILC (G3) with brisk mitotic activity (arrows).

In 1979, Martinez and Azzopardi characterized a series of 30 ILCs. They noted seven different growth patterns including dissociated growth, single files, trabecular, alveolar, solid, and plexiform growth, and admixed tubules [67]. Martinez and Azzopardi used the term classic ILC for the dissociated and single file growth pattern. “Single files” refers to chain-like cords of tumor cells. Ideally, tumor cells within single files are separated by tiny gaps (Figure 2). More compact cords are called trabeculae [67].

Single files lie between connective tissue fibers and elicit little or no desmoplastic reaction. Mammary ducts are encircled by tumor cells in a targetoid fashion. This has also been described as periparenchymal streaming [130]. Tumor cell density is low to moderate and satellite foci are common. Tumor cells accumulate at the border from connective to adipose tissue, or may appear to partially avoid infiltration into adipose tissue [20]. Together, these features are so characteristic that the definite diagnosis can almost be made at low power magnification. However, assessment at high power magnification is obligatory. At high power magnification, classic ILC features small tumor cells with scanty cytoplasm. Mitotic activity is low. Nuclei may vary in shape, but size and chromatin quality are fairly constant. Nuclei appear to be loose within the cytosol. A typical feature is that two tumor cells compress a third tumor cell lying in between. The compressed tumor cells experiences a biconcave or triangular compression of its nucleus (Figure 2). Histological grade G1 is associated with comparatively good short-term outcome, but the prognosis G2- and G3-differentiated cases is difficult to predict [61]. High grade ILC appears to be associated with worse outcome [61,73,89].

### 3.2. Related In Situ Lesions

Intra-acinar proliferation of dyscohesive epithelial cells in mammary lobules is termed lobular neoplasia (LN). LN is characterized by an uneven intraacinar cell distribution [20]. LN is nearly always E-cadherin-negative and shares DNA CN alterations and *CDH1* mutations with adjacent ILC [79,80,81,82]. LN is an umbrella term encompassing a spectrum of related, morphologically defined in situ lesions including atypical lobular hyperplasia (ALH), lobular carcinoma in situ (LCIS), lobular intraepithelial neoplasia (LIN), florid LCIS/LIN, and pleomorphic LCIS/LIN. LN is evident in approximately 50% of ILCs [49,50,51]. Transition from LCIS to ILC can be observed in some specimens, where LCIS cells breach trough the basal lamina (Figure 2) [131]. LN may also be found adjacent to tubular BC and columnar cell lesions of the mammary epithelium (“Rosen triad”) [132]. Pure LN without invasive BC may be encountered as an incidental finding in core needle biopsies (CNBs), reduction mammoplasties and benign lesions, such as fibroadenomas [133,134,135,136]. LN is often multifocal and bilateral [137].

Multiple nomenclatures have been proposed for LN. In 1952, Godwin reported on a female diagnosed with pure LCIS in an excision biopsy. She received no further treatment and succumbed to metastatic ILC 15 years later [138]. This report consolidated the notion that LCIS is an obligate precursor of ILC and a fatal disease, if not treated by radical mastectomy [138]. In 1974, Wheeler reported on patients diagnosed with LCIS, who did not undergo mastectomy. In an up to 25 years follow-up, only 1/25 (4%) patients developed ipsilateral ILC [139]. Similar findings were reported by Haagensen et al. and Rosen et al. in 1978 [6,140]. Subsequent studies confirmed that LCIS is a non-obligate precursor of ILC and a marker of increased ipsi- and contralateral BC risk (Appendix A, Table A3) [137,141,142,143,144,145,146,147,148,149,150,151,152]. Haagensen et al. viewed LCIS as a benign lesion and changed its name to lobular neoplasia (LN) [140]. To prevent over-treatment, LN of diminutive extent and bland cytomorphology was termed atypical lobular hyperplasia (ALH) [140]. Currently, ALH is defined as LN affecting and distending <50% of the acini in a terminal ductolobular unit (TDLU), with or without pagetoid extension in terminal ducts [19,151]. LCIS is defined as LN affecting and distending >50% of the acini in a TDLU [19,151]. This cutoff is arbitrary and inter-observer agreement is limited [153]. Bratthauer and Tavassoli have proposed an alternative sub-classification and used the term lobular intraepithelial neoplasia (LIN) as a synonym for LN and graded these lesions in a 3-tiered manner as LIN1-3, according to the degree of acinar distention (none, slight, maximal) [154]. The Bratthauer scheme was adopted in many countries, but it is not endorsed by the current WHO classification [153,155].

More importantly, LN may develop high grade nuclear atypia and bulky distention of TDLUs (defined as >50 “layers” of cells) [130,156,157,158]. Both features can be associated with comedo-necrosis and calcifications, which is otherwise rare in LN. These variants of LN are termed pleomorphic LCIS/LIN and florid LCIS/LIN [130,156,157]. They are associated with more complex genetic alterations and higher risk of synchronous and metachronous ILC [149,159,160,161,162,163,164,165,166,167]. Interestingly, comedo-necrosis has slightly different morphological features in LCIS and high grade ductal carcinomas in situ (DCIS). In DCIS, comedo-necrosis tends to be compact, with well-defined margins (plug-like necrosis). In LCIS, due to the lack of cell cohesion, comedo-necrosis is often fragmented and becomes haphazardly intermingled with LCIS cells (grid-like necrosis) [155]. Furthermore, pleomorphic LCIS/LIN is also characterized by an increased number of bi- and multinucleated tumor cells (≥5 per high power field), and lack of growth suppression in the center of distended acini (no Ki67 gradient towards the center), which are ancillary criteria for the diagnosis of pleomorphic LCIS/LIN [153,168]. The risk of progression of LCIS to ILC remains a matter of debate. We among others have also proposed to appreciate a Ki67 index of >10% to identify LCIS lesions at higher risk of progression [153,169,170]. This may serve as an additional criterion for choosing the most appropriate therapeutic strategy.

In EU national mammography screening programs, histologic diagnoses are translated into clinical intervention directives using the standardized B classification scheme [171]. Pleomorphic LCIS/LIN and florid LCIS/LIN are mostly classified as B5a lesions (in situ malignancy), while ALH and classic LCIS/LIN are classified as B3 lesions (uncertain malignant potential) [133,134,158,172]. Florid LCIS/LIN is classified as a B4 lesions (suspicious for malignancy) in the United Kingdom [158,172]. B5a and B4 lesions warrant surgical excision, while B3 lesions can be compatible with conservative management [134,173]. Detailed correlation of histologic and radiologic findings is always necessary, as most LN lesions are radiologically occult (except for florid and pleomorphic LCIS/LIN) and therefore may not always represent the lesion that prompted needle biopsy. The European B-classification system has successfully standardized and streamlined surgical intervention decisions for LN [134].

### 3.3. Immunohistochemical Features

**Figure 3:**
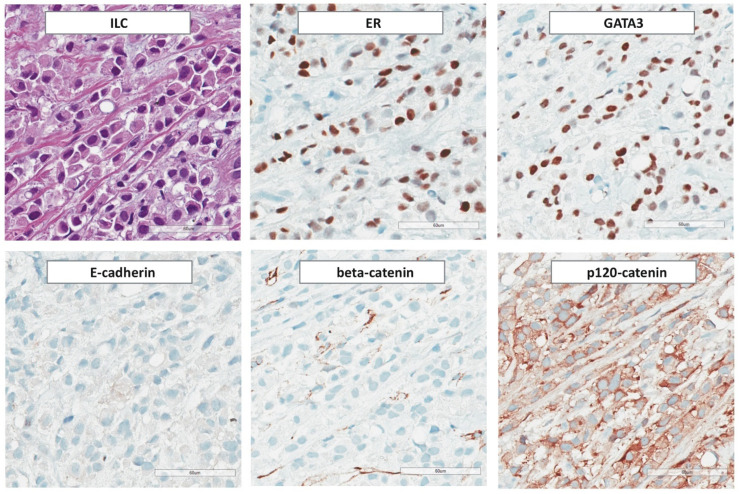
Immunohistochemical features of ILC. From Photomicrographs (×200 magnification) illustrate: expression of ER and GATA3, loss of E-cadherin and β-catenin, and aberrant cytoplasmic localization of p120-catenin.

ILC is nearly always ER-positive and progesterone receptor (PR)-positive (Figure 3). Overexpression and/or amplification of HER2 is rare (3–13%) [20,174,175]. However, HER2 overexpression and/or amplification occurs in a subset of pleomorphic ILCs (see Section 4.10) [176,177]. Activating mutations of *HER2/ERBB2* or *HER3*/*ERBB3* occur in approximately 5% of primary ILCs and in up to 18% and 15% of relapsed and grade 3 ILCs, respectively [43,117,161,178,179,180,181,182]. However, *HER2/ERBB2* mutation is not consistently associated with overexpression and is thus not detectable by immunohistochemical HER2 assessment according to ASCO/CAP guidelines [183,184]. Androgen receptor (AR) is positive in approximately 20% of cases [61]. An ER/PR/HER2-triple-negative immunophenotype is observed in 2–9% of ILCs [62,70,114,185]. However, the tumorbiology of triple-negative ILC may differ significantly from triple-negative BC (TNBC) of NST [186,187]. Conversely, ILCs account for 1–3% of all triple-negative BCs [188,189,190]. Nearly all triple-negative ILCs in Caucasian women are AR-positive (>90%) [187]. ILC retains luminal differentiation, even if the immunophenotype is ER/PR/HER2-triple-negative and AR-negative [186,190]. Triple-negative ILCs may harbor *HER2/ERBB2* or *HER3*/*ERBB3* mutations and have recently been associated with hotspot mutations in *ESRRA*, an orphan nuclear receptor with structural homology to ER [187]. Nuclear accumulation of p53 is rare in ILC and is associated with pleomorphic histology (see below). The median Ki67 index of classic ILC is approximately 10% [61,191,192,193,194,195,196]. Ki67 is prognostic in ILC, at least in univariate analyses [61,191,192,193,194,195,196].

Loss of E-cadherin, as determined by IHC, is observed in 55–100% of cases (Appendix A, Table A2) [9,10,11,12,41,42,43,59,61,82,88,90,99,100,101,102,103,104,105,106,107,108,109,110,111,112,113,114,115,116,117,118,119]. Aberrant fragmented, cytoplasmic or nuclear E-cadherin staining has been reported in as much as 16% and 35% of ILCs, respectively [109,124,197]. Aberrant nuclear staining appears to be a specific feature of a certain anti-E-cadherin antibody (clone-36) and is not observed, if immunohistochemistry is performed with other anti-E-cadherin antibodies (EP700Y, NCH-38, ECH-6) [197]. Partial cross-reactivity of the anti-E-cadherin antibody clone 4A2C7 with P-cadherin has been suggested [198]. This has highlighted the importance for detailed knowledge about specific staining properties of anti-E-cadherin antibodies and quality control measures. To this end, round robin tests for E-cadherin IHC have been conducted by the nordic immunohistochemical quality control program (NordiQC) [199]. Round robin test performance profiles of 25 different, commercially available anti-E-cadherin antibody formulations are available at the NordiQC webpage. For instance, a poor-signal-to noise ratio and/or false-positive E-cadherin-staining was noted in an increased proportion of laboratories using the anti-E-cadherin clone EP700Y.

E-cadherin is a component of AJs. β-catenin binds to the cytoplasmic domain of E-cadherin and links AJs to the cytoskeleton through α-catenin [92]. Loss of E-cadherin is accompanied by loss of β-catenin and cytoplasmic and/or nuclear localization of p120-catenin [15,100]. Focal upregulation of P-cadherin, an alternate type I cadherin, is not uncommon (approximately 8–16%) (see Section 4.6 and Section 5.2) [59,200,201]. Epithelial-to-mesenchymal transition (EMT) markers, such as Vimentin, N-cadherin, and Twist, are not expressed [115]. ILC is nearly always positive for low molecular weight cytokeratins CK7 and CK8/18 [175,202,203]. Ring-like perinuclear immunoreactivity for CK8 may occur [203]. Expression of high molecular weight (basal) cytokeratins CK5/6 and CK5/14 has been reported for 0–17% of cases [175,204,205,206]. In our experience, ILC is almost exclusively CK5/14-negative [114]. ILC is positive for luminal differentiation markers, including GATA3 [207,208]. Apocrine differentiation is observed in a subset of pleomorphic ILCs and is inherent to histiocytoid ILC (see below) [209]. ILC with apocrine differentiation is regularly ER/PR-negative, AR-positive, and exhibits enhanced expression of the prolactin inducible protein GCDFP-15 [210]. Overexpression of HER2 is also common in ILC with apocrine differentiation [209,211]. Approximately 80% of ILCs are positive for transcription factor AP2-β (TFAP2B) [39,212]. TFAP2B is a diagnostic marker for alveolar rhabdomyosarcoma (aRMS) and has been reported to mediate anti-apoptotic signals in aRMS [213,214]. However, TFAP2B is also expressed in LCIS and ILC [39,212]. Strong immunoreactivity for insulin-like growth factor IGF-1 is comparatively rare in BC of NST but common in ILC (>50% of cases) [215]. This is consistent with enhanced *IGF1* and *IGFR1* mRNA expression in ILC and with the concept that loss of E-cadherin uncouples contact inhibition and induces autocrine stimulation of growth factor receptor pathways [17,18,39]. Moreover, expression of the growth hormone-releasing hormone receptor (GHRH-R) has been associated with ILC and BCs with apocrine differentiation [216,217]. Expression of PD-L1 in ILC tumor cells (SP142 antibody) has been reported for up to 17% of cases [218]. The relevance of this finding remains to be demonstrated.

## 4. Histologic ILC Variants

### 4.1. Relevance of ILC Variants

**Figure 4:**
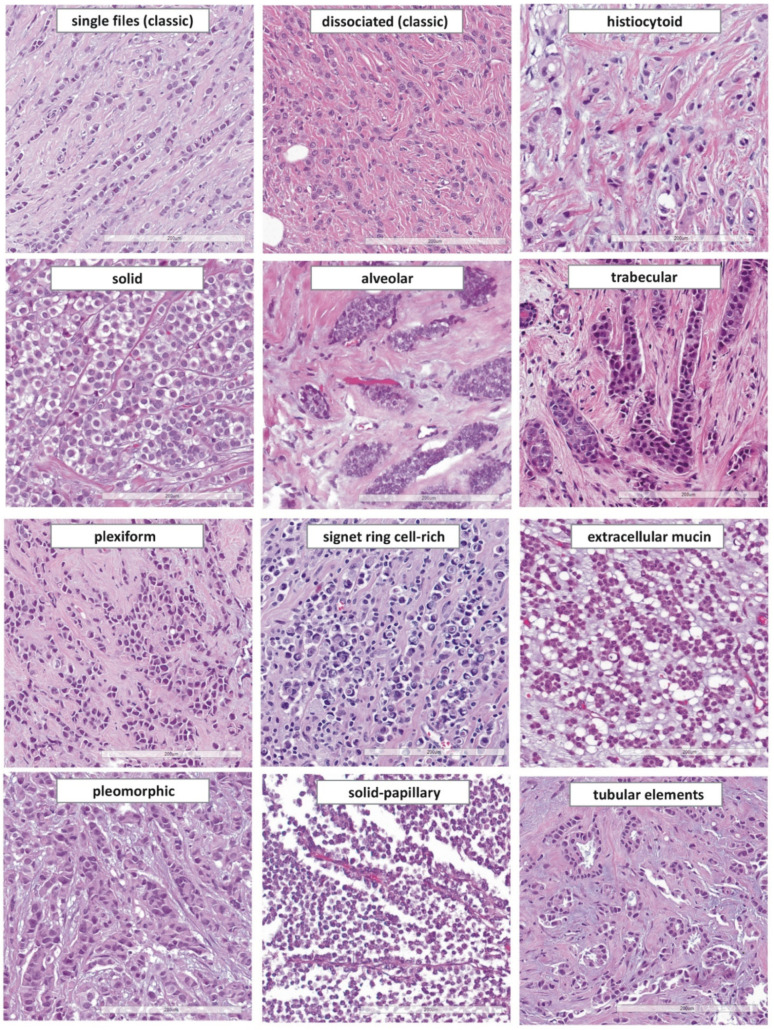
Histomorphology of ILC variants reported in the scientific literature. From top left to lower right: single file growth pattern (classical ILC), dissociated growth pattern (classical ILC), histiocytoid ILC, solid ILC, alveolar growth pattern, trabecular growth pattern, plexiform growth pattern, signet ring cell-rich ILC, ILC with extracellular mucin, pleomorphic ILC, solid-papillary ILC, ILC with tubular elements (×200 magnification).

ILC variants account for up to 70% of ILC cases (Appendix A, Table A4) [43,46,61,62,63,64,65,66]. The WHO classification of tumors of the breast (5th edition) mentions four different ILC variants (solid, alveolar, pleomorphic, tubulolobular) as well as histiocytoid differentiation and signet ring cell morphology [19]. However, more than 10 different histologic variants have been described in the scientific literature (see Section 4.2, Section 4.3, Section 4.4, Section 4.5, Section 4.6, Section 4.7, Section 4.8, Section 4.9, Section 4.10, Section 4.11, Section 4.12 and Section 4.13) (Figure 4). Some variants are named for their growth pattern, such as solid ILC. Other variants are named for cytologic features, such as pleomorphic ILC. Some variants are associated with distinct molecular alterations and slightly different clinical outcome [43,62,219]. The list of ILC variants below is not intended for splitting the diagnosis of ILC into a large number of subcategories, but it gives an overview of previously reported morphological variants. It may help to recognize given tumors as belonging to the lumped category of ILC, of which solid and pleomorphic variants are two distinct non-classical forms covered by the current WHO classification, along with tubulolobular carcinoma, which the authors believe belongs to non-lobular BCs, or to BCs with a heterogenous tumor biology.

### 4.2. Histiocytoid ILC

Histiocytoid ILC was first described by Hood et al. in 1973 (Figure 4) [220]. These authors reported a series of 13 female patients with BC metastases in the eyelid. The 12 metastases included one overt lobular BC (single file growth pattern) and eight cases described as histiocytoid cancer. The histiocytoid cases displayed few dissociated tumor cells and few single files, nuclei with little atypia and ample ground glass cytoplasm. Some of these cases were initially confused with benign lesions (including granular cell tumor and granulation tissue). Hood et al. were convinced that these histiocytoid carcinomas were metastatic ILC, but this was explained only in the discussion section of their article [220]. At that time, the diagnosis of ILC was still not easily made without evidence of LCIS [56]. Accordingly, it was difficult to establish the diagnosis of metastatic ILC, without the primary tumors at hand for comparison. Today, the study of Hood et al. is rarely cited. The specific propensity of ILC to metastasize to the eyelid and orbital adipose tissue was rediscovered forty-two years later by Raap et al. [31]. Following Hood’s article, two cases of primary histiocytoid ILC with adjacent LCIS were reported by Eusebi et al. and two further cases were reported by Walford et al. [210,221]. In these cases, adjacent LCIS displayed a wide morphological spectrum ranging from classical LCIS to acini replaced by histiocytoid cells [221]. Strikingly one of the two patients described by Walford et al., developed a metachronous metastasis of histiocytoid ILC to the eyelid [221]. Both studies associated histiocytoid ILC with apocrine differentiation. Later studies confirmed the lack of E-cadherin expression [222,223]. Histiocytoid ILC is mostly ER/PR-negative [224]. Expression of AR and GCDFP15 and overexpression of HER2 is common [211,223,224]. IHC for cytokeratins (CK7, CK8/18) and histiocytic cell markers (PGM1) is necessary to confirm the epithelial origin of tumor cells [225].

### 4.3. Solid ILC and Solid-Papillary ILC

Solid ILC was first described by Fechner et al. in 1975 (Figure 4) [66]. Fechner reported on invasive BCs associated with LCIS. His series included 6 cases, which displayed the typical lobular cytomorphology but grew in solid sheets [66]. This variant has also been called confluent ILC [226]. Solid ILC tends to have a higher histological grade and a higher Ki67 index. Mutation of *ARID1A* and *TP53* and CN gain of *ESR1* are more common in solid ILC [43]. Solid ILC has been associated with worse overall survival [62]. In CNBs, the differential diagnosis between solid ILC and mammary primary non-Hodgkin lymphoma is sometimes challenging, when mitoses are numerous. The latter diagnosis should be ruled out through ancillary IHC studies.

Solid-papillary ILC was first described by Rakha et al. and Christgen et al. in 2016 and 2017, respectively [227,228]. Both groups described BCs, which were initially classified as encapsulated papillary breast cancer in CNBs. The resection specimens revealed well-circumscribed tumors with a fibrous pseudocapsule, solid-papillary architecture, and satellite foci of classic ILC. IHC demonstrated loss of E-cadherin in both tumor components. Satellite foci and solid-papillary tumor tissue harbored identical *CDH1*/E-cadherin mutations and nearly identical DNA CN alterations [228]. Accordingly, these cases were classified as ILC with solid-papillary growth pattern [227,228]. Another solid-papillary-like ILC has been reported by Motanagh et al. [229]. Intratumoral cystic spaces and fibrovascular cores covered with tumor cells distinguish solid-papillary ILC from solid ILC, as described by Fechner et al. (Figure 4) [227,228,229]. The biological differences between these two variants are probably limited, if any. Solid ILC with papillary features may be an alternative terminus for these tumors. However, in CNBs, the solid-papillary growth pattern of ILC can be a challenge and may raise the differential diagnosis of a papillary neoplasm [230].

### 4.4. Signet Ring Cell-Rich ILC

Small intracytoplasmic lumina in ILC cells (without displacement of nuclei) were first described by Gad and Azzopardi in 1975 (Figure 4) [231]. Larger intracytoplasmic mucin vacuoles (with displacement of nuclei, corresponding to signet ring cells) were first described by Steinbrecher and Silverberg in 1976 [232]. These authors characterized ILCs with foci of >50 signet ring cells per high power field [232]. Contrary to mucinous BC, no extracellular mucin was noted [232,233]. Electron microcopy has shown microvilli-like structures in the inner membrane of these vacuoles [234].

### 4.5. Tubulolobular BC—Possibly Not an ILC Variant

Tubulolobular BC was first described by Fisher et al. in 1977 [235]. Fisher et al. reported a series of 24 BCs, which exhibited small tubules and cords of neoplastic cells reminiscent of ILC [235]. In their paper, the authors suggested these tumors had overlapping features of tubular and lobular BC. Their classification as one or the other was considered a philosophical matter (id est, giving more priority to tubules or to lobular-like growth pattern). However, these authors favored an interpretation as lobular BC on the basis of worse outcome than that of pure tubular BC, and they were possibly wrong as explained below. In 1979, Martinez and Azzopardi noted focal tubules in otherwise classic ILCs and carefully insinuated that these cases may represent pure ILCs [67]. Dixon et al. excluded tubulolobular BCs from ILC [236]. In 2004, Wheeler et al. reported a systematic immunophenotypic characterization of 27 tubulolobular BCs [237]. Strikingly, not a single case showed loss of E-cadherin expression [237]. Wheeler et al. concluded that the uniform and strong expression of E-cadherin in tubulolobular BC supports a ductal not a lobular differention [237]. Subsequently, Kuroda et al. and Esposito et al. confirmed E-cadherin expression in 12/16 and 19/19 tubulolobular BCs [238,239]. Esposito et al. also demonstrated regular β-catenin and p120-catenin expression. Subsequently, it was proposed that tubulolobular BC should be termed ductal BC with tubulolobular growth pattern [239]. This view was also supported by three-dimensional reconstruction of tubulolobular BC from cytokeratin-stained serial sections. Tumor cell cords were shown to correspond to long solid tails at the angular end of tear drop-shaped tubules [240]. However, tubulolobular BC is listed as an ILC variant in the current WHO classification [19]. The studies of Wheeler et al., Kuroda et al., and Esposito et al. are not cited in this context in the current WHO classification [19]. Some pathologists apply the term tubulolobular to E-cadherin-positive BCs, which mimic some single files. Other pathologists apply this term to genuine E-cadherin-negative ILCs with some E-cadherin-negative tubules. The latter constellation has recently been termed ILC with tubular elements (see below) [59]. The general perception of tubulolobular BC by most of the authors of this review is that the limited evidence against this being an ILC variant is greater than the evidence favoring its listing under ILC.

### 4.6. ILC with Tubular Elements

In 2020, three authors of the present review described ILC with tubular elements as another ILC variant (Figure 4) [59]. A series of 13 ILCs featuring non-cohesive tumor cells mixed with cohesive tubular elements was subjected to a molecular characterization [59]. All cases were E-cadherin-negative. Loss of E-cadherin expression distinguished these cases from E-cadherin-positive tubulolobular BC, as defined by Wheeler et al. [237,238,239]. DNA sequencing confirmed *CDH1*/E-cadherin mutations [59]. Noncohesive tumor cells were E-cadherin-negative and β-catenin-negative, while admixed tubular elements were E-cadherin-negative but β-catenin-positive. Focally retained β-catenin expression indicated rescue of AJs. Accordingly, these cases were screened for alternate cadherins. This revealed P-cadherin expression in tubular elements of 12/13 cases studied [59]. E-cadherin to P-cadherin switching has provided a molecular explanation for tubule formation in *CDH1*-deficient ILC. Studies in our laboratory now show that reconstitution of P-cadherin in an ILC cell line restores AJs and the formation of epithelial-like coherent sheets (Derksen et al.; unpublished data). To avoid confusion with tubulolobular BC (E-cadherin-positive), these cases were termed ILC with tubular elements (E-cadherin-negative and P-cadherin-positive) (Appendix A, Table A5) [59]. Further studies on P-cadherin expression in ILCs with tubular elements are warranted.

### 4.7. Alveolar ILC

Alveolar ILC was first described by Martinez and Azzopardi in 1979 (Figure 4) [67]. This growth pattern is characterized by loose, globular aggregates of >20 tumor cells, separated by thin bands of collagenous fibrosis [67]. The alveolar growth pattern can be mistaken for foci of LCIS. However, alveolar aggregates are invasive. Alveolar ILC is enriched in cases with CN gain on chromosome 11q13.3 (*CCND1*) and 11q14 (*PAK1*) [43]. Martinez and Azzopardi discussed that alveolar ILC is either an “attempt to reproduce” acinar structures, or a “phase” before ILC cells finally lose cohesion [67]. Polymorphous adenocarcinoma of the breast, a rare tumor similar to polymorphous adenocarcinoma of the salivary gland, is a potential differential diagnosis for alveolar and trabecular ILC. However, polymorphous carcinoma is triple-negative, BCL-2-positive, and E-cadherin-positive, and may pursue an aggressive course in young patients [241].

### 4.8. Trabecular and Plexiform ILC

Trabecular ILC was first described by Martinez and Azzopardi in 1979 (Figure 4) [67]. They distinguished between growth in single files, one-cell thick trabeculae, two-cell thick trabeculae, and three-cell thick trabeculae. One-cell thick trabeculae were described as more compact compared to single files. The authors admitted that this was subjective [67]. Trabecular ILC is not listed as an ILC variant in the current WHO classification [19]. Trabecular ILC accounts for 0–20% of ILC cases in published series [43,46,62]. Presumably, many pathologists include this morphology within classic ILC. In their seminal work on ILC, Martinez and Azzopardi also mentioned a plexiform pattern [67]. Paradoxically, none of their 30 ILCs was actually assigned to this growth pattern. In our experience, some ILCs are perfectly described by the term plexiform. These tumors grow in ragged clusters with loose anastomoses, which form a bizarre plexus (Figure 4).

### 4.9. Mixed Non-Classical ILC

Dixon et al. investigated the prognosis of ILC variants. In their study, which was published in 1982, ILCs were subclassified as classic, solid, alveolar and mixed non-classical [236]. Mixed/non-classical ILC included cases with several ILC variant growth pattern, trabecular growth and/or increased nuclear pleomorphism. Mixed non-classical ILCs accounts for 14–34% of ILCs in recent series [43,46,64]. Mixed/non-classical ILC must not be confused with mixed BC (NST/ILC) (see Section 2.2).

### 4.10. Pleomorphic ILC

Pleomorphic ILC is a de-differentiated variant and accounts for no more than 5% of ILCs [20,61,130]. ILCs with pronounced nuclear atypia were initially included within mixed non-classical ILC by Dixon et al. [236]. In the 1980s, a morphological description of pleomorphic ILC was provided by Page et al. [242]. In the 1990s, Eusebi et al., Weidner et al. and Bentz et al. reported on the dismal prognosis of pleomorphic ILC [130,243,244]. However, standardization of diagnostic criteria has remained a challenge. Weidner et al., proposed that classic ILC is only well-differentiated (nuclear grade 1). Subsequently, Weidner et al. defined pleomorphic ILC as all ILCs with greater nuclear pleomorphism than in classic ILC. In their study, pleomorphic ILCs were mostly histological grade 2 (87%) and only a single case was described as nuclear grade 3 [243]. Eusebi et al. defined pleomorphic ILC as ILC with large, lobulated, indented, and hyperchromatic nuclei, abundant eosinophilic or granular cytoplasm (indicative of apocrine differentiation), and/or rhabdomyoblastoid appearance [130]. These features are associated with increased mitotic activity (Figure 2 and Figure 4). This implies that pleomorphic ILC, as defined by Eusebi et al., was mostly histological grade 3 (modified Bloom–Scarf–Richardson score 3 + 3 + 2 = 8, G3). The 3.5-year overall survival rates reported by Weidner et al. and Eusebi et al. were approximately 75% and 40% [130,243]. This supports the view that pleomorphic ILCs, as described by Weidner et al. and Eusebi et al., were not quite congruent. Rakha et al. defined pleomorphic ILC as ILC with nuclear grade 3, irrespective of cell shape [245]. Monhollen et al. defined pleomorphic ILC as ILC with loss of E-cadherin and nuclei 4 × larger than a lymphocyte (nuclear diameter ≥18 µm) [177]. This cutoff has recently been included in the current WHO classification [19]. Experienced pathologists achieve fair interobserver agreement for the diagnosis of pleomorphic ILC [153]. Importantly, nuclear compression artifacts, which reflect tumor cell motility rather than dedifferentiation, should not prompt classification as pleomorphic ILC, unless accompanied by brisk mitotic activity, severely abnormal chromatin, and/or increased number of bi- and multinucleated tumor cells (≥5 per high power field) (Figure 2 and Figure 4) [153,168].

Pleomorphic ILC has escaped from the low-grade progression pathway associated with classic ILC [20,176]. Gene expression profiling has revealed few differences between classic and pleomorphic ILC [246]. However, pleomorphic ILC is characterized by more complex DNA CN alterations and may show loss of ER and/or overexpression of HER2 (Appendix A, Table A6) [116,117,176,177,245,247,248,249,250,251,252,253,254,255,256]. *TP53* mutation is rare in classic ILC (<10%), but occurs in 11–42% of pleomorphic ILCs [117,257]. P53-dependent suppression of the PI3K/AKT pathway may govern the transition from classic to pleomorphic ILC [258]. Pleomorphic ILC is also associated with high oncotype DX recurrence scores (RS > 25), increased genomic grade index, FER kinase expression, altered DNA methylation pattern, and mutation of *IRS2* and *IGFR1* [61,116,259,260,261]. Triple-negative pleomorphic ILC has been associated with mutation of *ESRRA* (76%) [187]. Conflicting data exist regarding the frequency of *HER2/ERBB2* mutation in pleomorphic ILC (7–26%) (Appendix A, Table A6) [116,117,252,255,256]. HER1/EGFR or HER2 kinase inhibitors (lapatinib, neratinib) are effective in metastatic ILC harboring *HER2*/*ERBB2* mutations [3,262,263,264,265]. The prognosis of pleomorphic ILC treated with modern regimens is probably not as fatal as in the 1990s [61,177,266,267]. Haque et al., reported a five-year overall survival of 76.7% for pleomorphic ILC (including triple-negative and HER2-positive cases) [267]. Pleomorphic ILC was not associated with worse overall survival, if cases were corrected for the HER2 status [267]. The prospective WSG PlanB trial reported a five-year disease-free survival of 78.8% for patients with hormone receptor-positive/HER2-negative early pleomorphic ILC [61].

### 4.11. ILC with Extracellular Mucin

ILC with extracellular mucin was first described by Rosa et al. in 2009 (Figure 4) [268]. Rosa et al. reported on a BC composed of 80% classic ILC and 20% signet ring cells floating in pools of extracellular mucin. Both tumor components were E-cadherin-negative [268]. Similar cases were reported by Yu et al. and Gomez-Macias et al. [269,270]. BCK-4 is a human BC cell line derived from an ILC with extracellular mucin [271,272]. Estrogen withdrawal decreases tumor growth but accelerates mucin secretion in BCK-4 xenograft mouse models [271]. BCs of NST and ILC are typically MUC1-positive (91%), but rarely MUC2-positive (8%) [273]. Cserni and colleagues compiled a series of 8 ILCs with extracellular mucin [274]. Coexpression of MUC1 and MUC2 was noted in 7/8 cases, suggesting that the secreted mucin MUC2 is involved in the morphogenesis of this ILC variant [274,275]. The diagnosis of ILC with extracellular mucin may pose challenges on CNB because it is always associated with a non-mucinous ILC component which is not infrequently a solid component. Over 50% of the cases present with lymph-node involvement, HER2 amplification is reported in 12–40%, and high nuclear grade is also frequently reported [276]. Next generation sequencing interrogating the full coding sequences of 447 genes revealed *CDH1* mutations in 8/8 cases, and *TP53* and *PIK3CA* mutations were the most frequent alterations in cases that showed relapse [276].

### 4.12. ILC with Neuroendocrine Features

ILC with neuroendocrine features was first highlighted by Risaliti et al. in 1989 [277]. These ILCs feature nested growth, salt and pepper chromatin, and expression of one or more neuroendocrine markers, such as chromogranin A or synaptophysin [175,278,279,280].

### 4.13. ILC of the Diffuse Type

ILC of the diffuse type was proposed as a diagnostic category by Tot in 2003 [281]. Based on sub-gross morphology, Tot distinguished between unifocal, multifocal, and diffuse ILC. ILC of the diffuse type is not a histological variant, but refers to a spider web-like tumor distribution in the mammary gland [281]. ILCs with this distribution pattern account for approximately 28% of all ILC cases and are associated with nodal involvement and poorer overall survival [281,282,283].

## 5. Advances

### 5.1. Laminin and Pagetoid Extension of LCIS

Pagetoid extension of LCIS in mammary ducts was first described by Foote and Stewart [7]. Pagetoid extension refers to LCIS growing underneath the normal epithelium of terminal ducts. Warner described this as a collar around the epithelial cell layer [47]. Pagetoid extension is highly specific for and nearly universal in LCIS [139]. A recent study of genetically engineered mouse (GEM) models has revealed the molecular basis of this growth pattern [284].

Somatic inactivation of *Cdh1*/E-cadherin alone does not predispose mice to mammary tumors in conditional knockout GEM models [13,285,286]. Clearance of *Cdh1*/E-cadherin-deficient cells by cell death prevents tumor formation [285]. However, mouse ILC develops in compound conditional knockout GEM models, in which *Cdh1* inactivation is combined with activation/inactivation of an additional oncogene or tumor suppressor gene, such as *Trp*53, *Pten*, or *Pik3ca* [13,287,288,289,290,291]. Schipper et al. have combined *Cre* recombinase-induced *Cdh1* inactivation with induction of a reporter gene (GFP), which allowed to scrutinize the fate of *Cdh1*-deficient cells [284]. The authors reported that E-cadherin-deficient epithelial cells degenerate due to high motility and membrane blebbing. However, some E-cadherin-deficient cells migrated underneath the basal epithelial cell layer, where they formed stationary clusters. The basal lamina component laminin was suggested to protect E-cadherin-deficient cells from lethal hyper-motility by inhibition of RhoA activity [284]. Unfortunately, Schipper et al. have not included the term pagetoid LCIS in their article. Many pathologists have not taken notice of this interesting finding. It may explain a long known histomorphologic feature of human LCIS and links pagetoid extension of LCIS with the inactivation of E-cadherin [284].

### 5.2. E-cadherin to P-cadherin Switching and Tubular Elements

**Figure 5:**
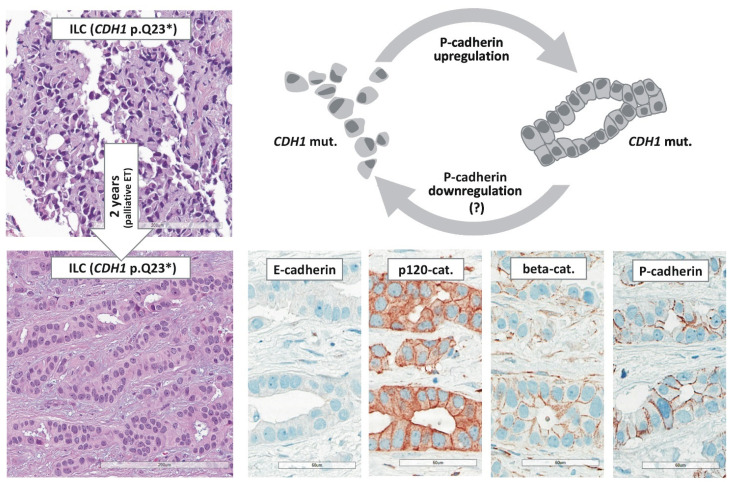
E-cadherin to P-cadherin switching in ILC with tubular elements [59]. Photomicrographs (×200 magnification) illustrate an ILC diagnosed by CNB (upper left) and the resection specimen after primary endocrine therapy (ET). Both specimens were E-cadherin-negative and harbored the same deleterious *CDH1* mutation (p.Q23*). Note that the resection specimens displayed tubular elements which were associated with expression of P-cadherin and β-catenin, while p120-catenin showed a partially aberrant cytoplasmic localization and a partially regular membranous localization. The cartoon illustrates that ILC cells form focal tubular elements by dynamic upregulation of P-cadherin (id est, E-cadherin to P-cadherin switching) [59].

In 1979, Martinez and Azzopardi emphasized that some ILCs display focal tubules [67]. Meanwhile, so-called tubulolobular BC has been disqualified as an E-cadherin-positive variant of ductal BC (id est, a mimic of ILC) [237,238,239,240]. Even so, many pathologists have remained supporters of the idea that focal tubules are compatible with the diagnosis of pure ILC [74,281,292]. A recent study revealed that E-cadherin to P-cadherin switching is the molecular determinant of such tubules in *CDH1*-deficent ILC (Figure 5) [59]. P-cadherin is encoded by *CDH3*. Both genes, *CDH1* and *CDH3*, map to chromosome 16q22.1 (*CDH3* is directly upstream of *CDH1*). *CDH1* and *CDH3* share 66% homology and nearly identical exon-intron structures [293,294]. From an evolutionary perspective, *CDH3*/P-cadherin arose by duplication of *CDH1*/E-cadherin in the carboniferous, some 300 million years ago [295]. E-cadherin and P-cadherin and their orthologs exert similar functions in different tissue compartments of various species. In birds, P-cadherin has adopted some of the roles dedicated to E-cadherin in mammals [296]. Cadherin switching is a physiological process in organ morphogenesis and cell differentiation [297]. Cadherin switching describes that cells shift to express different cadherins. A classic example is E-cadherin to N-cadherin switching, which regulates primitive streak formation [297]. In the normal mammary gland, E-cadherin expression is limited to the luminal epithelium, while P-cadherin expression is limited to the myoepithelial cell layer [298]. P-cadherin expression is common in triple-negative BC (approximately 40–80%), but less common in hormone-receptor-positive BC (approximately 10–23%) [59,197,201,298,299]. An in vitro cell model based on transient transfection of human BC cell lines indicates that P-cadherin can rescue AJ formation in the absence of E-cadherin [200]. Aberrant cadherin switching has been described in a variety of tumor entities [300]. However the relevance of E-cadherin to P-cadherin switching for ILC has just recently been recognized [59]. E-cadherin-negative ILC cells can activate weak to moderate P-cadherin expression to re-gain focal cell adhesion (Figure 5) [59]. This results in focal tubular elements in otherwise dyscohesive classic ILC [59]. Transient E-cadherin to P-cadherin switching may be involved in tumor dormancy in ILC [59].

### 5.3. ILC and the Microenvironment

Although it is clear that BC of NST and ILC are intrinsically different based on morphology, transcriptomics and proteomics, it is less clear if the composition of the ILC microenvironment has unique features [301,302,303]. Already in the early 1990s it was reported that ILC showed increased levels of integrin α1β1 and α6β1 expression [304]. In this context, the expression of the αv integrin and Thrombospondin-I (TSP1) were linked to ILC [305]. Using electron microscopy, it was found that ILC cells showed strong expression of plasma membrane-localized TSP1, which coincided with strong expression of the integrin subunits αv and α1 [306]. TSP1 is a secreted protein that associates with collagen-rich extracellular matrix (ECM) and possesses potent anti-angiogenic activity, a fact that is in apparent conflict with findings that ILC shows a higher level of microvascular density than BC of NST [215]. Interestingly, αv integrins expressed on mammary cells (αvβ3, αvβ5, αvβ6) are mostly restricted to the myoepithelial lineage, where they bind RGD-type ligands such as Vitronectin, Fibronectin, and Osteopontin. It is therefore surprising that a typical luminal ILC cell expresses myoepithelial markers like αv and α1, suggesting that it has acquired at least some basal-like characteristics [307]. It furthermore points towards a scenario where the anti-adhesive and haptotactic properties of TSP1, combined with the acquisition of myoepithelial traits and loss of E-cadherin, may favor single cell ameboid-type invasion modes [308]. For these typical invasion modes ILC may depend on lysyl oxidases (LOX), because a recent study showed that ILC cells rely on LOX-like 1 (LOXL1). LOXL1 is a secreted ECM modifier implicated in collagen cross-linking and integrin-mediated invasion processes, for proliferation and invasion [309].

The characteristic invasion mode of ILC, combined with specific ECM deposition could favor influx of specific inflammatory cells. Tumor-infiltrating lymphocytes (TILs) have become an important and trending topic in cancer biology, and it is evident from transcriptomic studies that ILC may harbor ‘immune’-enriched subtypes [42,45,302]. Research in GEM mouse models of ILC has already shown that IL17-producing γδ T-cells promote pulmonary and lymphatic dissemination through systemic, granulocyte colony-stimulating factor (G-CSF)-dependent expansion and polarization of neutrophils [310]. In human ILC, a study by Droeser et al. demonstrated that the influx of CD4-positive and FOXP3-positive T-cells was increased in high-risk BC of NST, but not in ILC [311]. Moreover, the authors found that the absolute number of TILs did not represent a major prognostic indicator in either BC of NST or ILC [311]. A separate study by Desmedt et al. showed that although TILs were associated with young age, lymph node involvement, high tumor cell proliferation and worse prognosis, the TIL percentages were substantially lower in ILC compared to BC of NST [44]. These findings have been independently confirmed by Tille et al., who demonstrated that in a cohort of 459 consecutive ILC cases, TILs were associated with younger age, larger tumors, lymph node involvement, HER2 amplification, multi-nucleation, and prominent nucleoli [46]. In this study, the authors showed that increased TILs also correlated with poorer invasive disease-free survival and overall survival and that ILCs with high TILs (stroma TILs > 5%) represented a minority of ILC cases (35%) [46].

Because of the correlations with worse survival and worse clinicopathological parameters, TIL influx is of particular interest in the context of modulation of immune checkpoints, which may provide options for antibody-based blockades targeting the pathway governed by, e.g., programmed cell death protein-1 (PD-1) and its ligand programmed cell death ligand 1 (PD-L1) [312]. In a study by Thompson et al., all ILCs contained at least some PD-L1-positive TILs (SP-142 antibody) [218]. Although the authors found that PD-L1 was expressed by tumor cells in 17% of ILCs, this expression did not correlate with immune infiltrate density, histological grade, ER or HER2 status [218]. In short, it appears that a subset of primary ILCs express PD-L1 on tumor cells and contain PD-L1-positive TILs, and it remains to be determined if these coincide with the ILC subsets that harbor increased TILs. Recently, an ILC-specific clinical trial has been commenced at the Netherlands Cancer Institute/Antoni van Leeuwenhoek Hospital that investigates the benefit of immune checkpoint blockade in ILC. This phase 2 trial, called GELATO (AssessinG Efficacy of Carboplatin and ATezOlizumab in Metastatic Lobular Breast Cancer; NCT03147040), will analyze the response to a combination of immunotherapy and chemotherapy in patients with metastatic ILC.

Cancer-associated fibroblasts (CAFs) are one of the most abundant non-cancer cell populations of the tumor microenvironment. Integrated analyses of different CAF markers (SMA, CD29, FAP, FSP1, PDGFRβ, CAV1) have identified four recurrent CAF subtypes (CAF-S1 to CAF-S4) across human cancers [313,314]. Although the myofibroblastic subsets (SMA-positive) CAF-S1 and CAF-S4 are strictly detected in tumors, the CAF-S2 and CAF-S3 (SMA-negative) subsets are observed both in tumors and normal tissues, suggesting these latter are reminiscent of normal fibroblasts. In addition, CAF1 have clearly demonstrated an immunosuppressive function. Conflicting data exist regarding immunophenotypic properties of fibroblasts in the ILC tumor stroma. ILC often show minimal stroma reaction at the invasive tumor front, which leads to frequent clinico-radiological underestimation of the actual tumor size. The few available CAF studies, based on IHC for selected single markers, revealed that CAF markers are differentially expressed in ILC, with high levels of FAP, FSP1 and PDGFRβ. In some ILCs (15%), stroma fibroblast may not convert to a CD34-negative phenotype, but may retain CD34 expression [303,315]. Notably, the expression levels of some of these stroma cell surface proteins may correlate with nodal stage (PDGFRα) or disease-free survival (FSP1, Podoplanin) [215,316]. We believe that the interplay between CAFs and TILs in ILC remains to be further elucidated.

## 6. Conclusions

Modern tumor classifications have evolved from and are based on histomorphology. ILC has been one of the first tumor types recognized as a special entity based on histomorphology [7]. ILC has also been one of the first tumor types associated with a specific tumor suppressor gene, namely, *CDH1*/E-cadherin [11]. In the clinic, special treatment strategies have been established for triple-negative, HER2-positive, and ER-positive BC. Treatment optimization for patients diagnosed with ILC is a new field of clinical research. Several new clinical trials, such as the GELATO (NCT03147040), the ROlo (NCT03620643), and the ROSALINE (NCT04551495) trials assess the efficacy of new treatment strategies for patients diagnosed with ILC. The distinct histomorphology of ILC clearly reflects a special tumor biology. The spectrum of tumors collectively classified as ILC deserves greater attention as a special tumor entity in patient care and cancer research. This review has briefly summarized the developments in the histomorphologic assessment of ILC from the 1940s until today. New ILC variants have just recently been recognized [228,274]. The pagetoid growth pattern of LCIS has been linked to the basal lamina component laminin [284]. E-cadherin to P-cadherin switching is a new mechanisms, that explains part of the morphological plasticity of ILC [59]. Recent studies have begun the explore the role of TILs and the tumor microenvironment in ILC [44,45,46]. The specificities of triple-negative ILC with the identification of *ESRRA* gene mutation may open a path to special therapeutic strategies [187]. Concepts of ILC are changing over time. Currently, ILC is still widely defined as a growth pattern [19]. At the same time, ILC is increasingly perceived as a distinct morpho-molecular BC entity. Refined criteria for ILC within a framework of a morpho-molecular entity have not yet been established but this appears achievable. Until then, translational research on ILC may have to face the challenges of tumor tissue review and standardization of inclusion criteria [61,78]. The goal of improved diagnosis and treatment for patients with ILC is worth these efforts.

## Data Availability

Not applicable.

## Appendix A

**Table A1:** Proportion of mixed BC (NST/ILC).

		BC			
	Cases	NST	Mixed (NST/ILC)	ILC	Ref.
Christgen et al. (2020) ^a^	2515	86%	<1%	14%	[61]
Flores-Diaz et al. (2019) ^b^	4733	86%	4%	10%	[70]
Ciriello et al. (2015) ^c^	705	70%	12%	18%	[42]
Braunstein et al. (2015) ^d^	998	74%	18%	8%	[71]
Bharat et al. (2009) ^e^	4336	83%	6%	11%	[69]
Louwman et al. (2007) ^f^	145,180	84%	4%	12%	[68]
Sastre-Garau et al. (1996) ^g^	11,036	91%	2%	7%	[27]
Martinez and Azzopardi (1979) ^h^	203	80%	5%	15%	[67]

Criteria for the classification of BC as mixed BC NST/ILC included ^a^ no specific statement in the published study [61]. According to personal communications, the following criteria were used: Presence of non-lobular and lobular growth pattern combined with loss of E-cadherin in the lobular growth pattern in one tumor. ^b^ No specific statement in the published study [70]. ^c^ Classification was based on local and central pathology assessment. Mixed BC (NST/ILC) was assumed, if local plus central subtype calls had the following constellations: ILC + NST, mixed + mixed, NST + ILC, or NST + mixed [42]. ^d^ Presence of features of both NST and ILC within one tumor [71]. ^e^ No statement in the published study [69]. ^f^ No specific statement in the published study. Nation-wide data; variable classification criteria cannot be ruled out [68]. ^g^ No specific statement in the published study [27]. ^h^ BC were classified as mixed BC NST/ILC, if the authors felt that they “could not state objectively whether an invasive carcinoma was of ductal, lobular, or mixed type” [67].

**Table A2:** Loss of E-cadherin and/or *CDH1* mutation in ILC.

Study	Cases	Loss of E-Cadherin	*CDH1* Mutation	Ref.
Rinaldi et al. (2020)	611		79%	[119]
Christgen et al. (2020)	353	96%		[61]
Christgen et al. (2020)	13	100%	85%	[59]
Sokol et al. (2019)	180		77%	[118]
Christgen et al. (2019)	106	99%	41%	[117]
Zhu et al. (2018)	17		59%	[116]
Desmedt et al. (2016)	413	85%	65%	[43]
Sakr et al. (2016)	21	100%	66%	[82]
McCart-Reed et al. (2015)	148	76%		[115]
Ciriello et al. (2015)	127		63%	[42]
Christgen et al. (2013)	43	98%		[114]
Boyault et al. (2012)	12		58%	[113]
Koboldt et al. (2012)	36		83%	[112]
Lips et al. (2012)	75	85%		[111]
Ellis et al. (2012)	40		50%	[110]
Rakha et al. (2010)	239	84%		[109]
Zou et al. (2009)	20	100%		[108]
Bertucci et al. (2008)	21	86%	62%	[41]
Da Silva et al. (2008)	25	85%		[90]
Turashvili et al. (2007)	29	93%		[107]
Reis-Filho et al. (2006)	13	92%	15%	[106]
Caldeira et al. (2006)	5	100%		[105]
Qureshi et al. (2006)	49	90%		[104]
Sarrio et al. (2004)	69	78%		[103]
Sarrio et al. (2003)	51	76%	22%	[99]
Lei et al. (2002)	25		12%	[102]
Droufakou et al. (2001)	22	55%	27%	[88]
Huiping et al. (1999)	40	94%	15%	[101]
Leeuw et al. (1997)	38	84%	55%	[100]
Berx et al. (1996)	41	84%	56%	[12]
Berx et al. (1995)	7	86%	57%	[11]
Moll et al. (1993)	22	86%		[9]
Gamallo et al. (1993)	7	100%		[10]
median 2011–2020		97%	65%	
median 1993–2010		86%	27%	

**Table A3:** Metachronous BC in patients diagnosed with pure LN and treated without mastectomy.

Study	Cases	Histology	Surgery	Follow-Up	Metachronous Invasive Cancer	Metachronous Ipsilateral ILC	Ref.
Lo et al. (2018)	732	LCIS	n.a.	>9 yrs	10%	n.a.	[150]
DeBrot et al. (2017)	7	pleo. LCIS	loc. ex.	>5 yrs	n.a.	29%	[149]
King et al. (2015)	1004	LCIS	loc. ex.	>6 yrs	11%	3%	[137]
Cutui et al. (2015)	159	LCIS	loc. ex.	6 yrs	10%	2%	[148]
Hartmann et al. (2014)	327	ALH	var.	>12 yrs	18%	2%	[147]
To et al. (2014)	35	LCIS	var.	20 yrs	21%	n.a.	[146]
Provencher et al. (2012)	275	LN	CNB	5 yrs	n.a.	1% *	[145]
Aulmann et al. (2008)	88	LCIS	var.	>10 yrs	10%	6%	[144]
Chuba et al. (2005)	4853	LCIS	n.a.	n.a.	7%	<1%	[143]
Fisher et al. (2004)	180	LCIS	loc. ex.	12 yrs	10%	4%	[142]
Fisher et al. (1996)	182	LCIS	loc. ex.	5 yrs	3%	2%	[141]
Haagensen et al. (1978)	209	LCIS	var.	11–42 yrs	17%	6%	[140]
Rosen et al. (1978)	99	LCIS	loc. ex.	24 yrs	32%	8%	[6]
Wheeler et al. (1974)	25	LCIS	var.	7–25 yrs	4%	4%	[139]
McDivitt and Stewart (1967)	40	LCIS	loc. ex.	10 yrs	15%	n.a.	[152]
Godwin et al. (1952)	1	LCIS	loc. ex.	15 yrs	100%	100%	[138]

ALH, atypical lobular hyperplasia; CNB, core needle biopsy only; LCIS, lobular carcinoma in situ; LN, lobular neoplasia; loc. ex., local excision; pleo, pleomorphic; var., variable; yrs, years. * Ipsilateral ILC in the same quadrant of the breast.

**Table A4:** Proportion of ILC variants.

		ILC			
	Cases	Classic	Variants	Reported Histologies *	Ref.
Tille et al. (2020)	459	68%	32%	s, a, m	[46]
Christgen et al. (2020)	353	90%	10%	s, a, p, o	[61]
Desmedt et al. (2016)	413	48%	52%	s, t, a, m	[43]
Iorfida et al. (2012)	981	56%	44%	s, t, m	[62]
Orvieto et al. (2008)	530	57%	43%	s, a, p, tl, o	[63]
Rakha et al. (2008)	517	55%	45%	s, a, m, tl, o	[64]
Du Toit et al. (1989)	171	30%	70%	s, a, m, tl, o	[65]
Fechner et al. (1975)	26	77%	23%	s	[66]

* Reported histological ILC variants included: s, solid; t, trabecular; a, alveolar; m; mixed/non-classical; p, pleomorphic; tl, tubulolobular; o, others, including signet ring cell-rich, apocrine, histiocytoid.

**Table A5:** Suggested IHC work-up for the differential diagnosis in BCs with mixed-appearing morphology (modified according ref. [61]).

Diagnosis	Collision BC (NST + ILC)		Tubulo- Lobular BC		ILC with Tubular Elements	
Component	Dissociated	Tubules	Dissociated	Tubules	Dissociated	Tubules
E-cadherin	neg.	pos.	pos.	pos.	neg.	neg.
β-catenin	neg.	pos.	pos.	pos.	neg.	pos.
P-cadherin					neg.	pos.

**Table A6:** Features of pleomorphic ILC.

		IHC Markers				Mutation		
Study	Cases	ER pos.	HER2 pos.	p53 pos.	E-cad. neg.	*TP53*	*ERBB2*	Ref.
Riedlinger et al. (2021)	16	81%	25%			25%	19%	[256]
Rosa-Rosa et al. (2019)	27	74%	3%		85%		26%	[255]
Christgen et al. (2019)	27	88%	0%	4%	100%	11%	7%	[117]
Zhu et al. (2018)	17	81%	25%			12%	17%	[116]
Liu et al. (2018)	46	85%	9%					[254]
Ilic et al. (2016)	53	64%	18%	42%	61%			[253]
Lien et al. (2015)	21	46%	33%		100%		14%	[252]
Rakha et al. (2013)	16	86%	0%		82%			[245]
Monhollen et al. (2012)	26	82%	35%	42%	100%			[177]
Ercan et al. (2012)	19					42%		[257]
Simpson et al. (2008)	26	76%	14%	13%	100%			[176]
Palacios et al. (2003)	29				100%			[251]
Sneige et al. (2002)	14	100%	8%	29%	100%			[250]
Frolik et al. (2001)	30	93%	13%	3%				[249]
Radhi et al. (2000)	10	20%		80%				[248]
Middleton et al. (2000)	38	81%	81%	48%				[247]
